# The influence of extreme thermal stress on the physiological and psychological characteristics of young women who sporadically use the sauna: practical implications for the safe use of the sauna

**DOI:** 10.3389/fpubh.2023.1303804

**Published:** 2024-01-26

**Authors:** Robert Podstawski, Krzysztof Borysławski, Natalia Maja Józefacka, Jadwiga Snarska, Bożena Hinca, Elżbieta Biernat, Anna Podstawska

**Affiliations:** ^1^School of Public Health, University of Warmia and Mazury in Olsztyn, Olsztyn, Poland; ^2^Angelus Silesius University of Applied Sciences, Institute of Health, Wałbrzych, Poland; ^3^Institute of Psychology, Pedagogical University of Krakow, Krakow, Poland; ^4^Department of Physical Education and Sport, University of Gdańsk, Gdańsk, Poland; ^5^Collegium of World Economy, SGH Warsaw School of Economics, Warsaw, Poland; ^6^Independent Researcher, Olsztyn, Poland

**Keywords:** female university students, Finnish sauna, different temperatures, mood, blood pressure, heart rate

## Abstract

**Background:**

Many individuals who use the sauna at a temperature of 120°C of higher are not aware of the negative consequences of extreme thermal stress. Despite extensive research into sauna use, the impact of extreme thermal stress on the physiological and psychological characteristics of sauna users have not been examined to date.

**Aim:**

The aim was to determine the effect of 20 min sauna sessions with a temperature of 80°C and 120°C on the physiological and psychological characteristics of women who sporadically visit the sauna.

**Methods:**

The study was conducted on 22 full-time female university students. Physical activity (PA) levels were evaluated with the Polish short version of the International Physical Activity Questionnaire (IPAQ). Anthropometric characteristics were measured before the first sauna session by the InBody270 body composition analyzer. Physiological parameters, including heart, energy expenditure, physical effort, and blood pressure (systolic blood pressure – SBP, and diastolic blood pressure – DBP), were assessed indirectly using Polar V800 heart rate monitors and the Omron M6 Comfort blood pressure monitor. The participants’ wellbeing was assessed with the Profile of Mood States (POMS) questionnaire. The presence of significant correlations between heat exhaustion and heat stress variables and syncope during the second sauna session was examined with the use of classification and regression trees (CRT) and the cross-validation technique.

**Results:**

Twenty-minute sauna sessions with a temperature of 80°C and 120°C induced a significant (*p* < 0.001) decrease in the values of SBP (excluding the temperature of 120°C), DBP, and body mass, as well as a significant increase in HR and forehead temperature. Exposure to a temperature of 80°C led to a significant (*p* < 0.001) increase in vigor with a simultaneous decrease in tension, depression, anger, fatigue, and confusion. In turn, sauna bathing at a temperature of 120°C had an opposite effect on the above mood parameters. Vomiting and confusion were the main predictors of syncope that occurred in some of the surveyed women.

**Conclusion:**

Excessive air temperature can induce symptoms characteristic of heat exhaustion and heat stress nausea, heavy sweating, fast weak or strong HR, high body temperature, and confusion. Therefore, sauna bathing at a temperature of 80°C can be recommended to women who sporadically use the sauna, whereas exposure to a temperature of 120°C is not advised in this group of sauna users. The present findings provide highly valuable inputs for managing wellness and SPA centers.

## 1 Introduction

Thermal stress resulting from exposure to high and low temperatures is often considered a challenge to human health and homeostasis. However, variable environmental conditions improve the physiological adaptation of the human body to heat and cold to a certain extent (1, 2). Heat stress has been researched more extensively than cold stress, but most studies indicate that both heat (3–9) and cold stress (10–14), as well as alternating heat/cold stress (15, 16) can deliver health benefits regardless of other lifestyle factors.

Finnish sauna is a typical example of heat therapy. Traditional Finnish sauna has been used for thousands of years for leisure, relaxation, and wellness to reduce the stress of everyday life (17, 18). To date, most studies have investigated traditional Finnish sauna sessions which consist of short-term (5–20 min) heat exposures to temperatures of 80°C–100°C and dry air (relative humidity of 10 to 20%), interspersed with periods of increased humidity when water is poured on hot rocks (19). Sauna bathing is “an ancient habit in both cold and warm climates” (20). These facts are relatively unknown to sauna users who generally view the sauna as short-term exposure to exceptionally high environmental temperatures. A sauna bath involves repetitions of alternating heat with cold, and it exerts bidirectional effects on the human body, depending on the hot and cold ratio and the intensity of heat and cold exposure. Unlike the warm-up phase, sudden exposure to cold activates the sympathetic nervous system and causes constriction of cutaneous blood vessels. Cold exposure generally induces opposite changes in the cardiovascular system than heat exposure: the heart rate (HR) decreases, whereas stroke volume and diastolic (DBP) and systolic (SBP) blood pressure increase (16, 21, 22).

There is considerable evidence to suggest that sauna baths can induce profound physiological effects (23–27). Regular sauna use has been found to improve the health-related quality of life (28). Increased frequency and duration of sauna bathing are inversely, strongly, and independently associated with fatal coronary heart disease and cardiovascular disease (CVD) events, all-cause mortality, and the risk of sudden cardiac death (SCD) (29). Emerging evidence suggests that sauna bathing improves cardiovascular health by decreasing arterial stiffness and BP, and improving selected blood-based biomarkers (30). Sauna baths have been linked to improved management of pain and symptoms associated with musculoskeletal disorders, including osteoarthritis, rheumatoid arthritis, and fibromyalgia (31, 32). Sauna bathing was also reported to improve mood (33, 34) and alleviate headaches (35). In regular users, the relaxing effects of sauna therapy combined with cold water immersion usually lead to a decrease in serum cortisol levels (27).

In the last decade, the influence of thermal stress on athletes has been investigated by numerous researchers who found that the final effect was determined by various factors. During far-infrared sauna (FIRS) sessions at moderate temperatures (35–50°C) and low humidity (25–35%), the deep penetration of infrared heat (approx. 3–4 cm into the adipose tissue and the neuromuscular system) contributes to the recovery of neuromuscular performance after maximal endurance training. FIRS bathing exerts a light load on the body and provides a comfortable and relaxing experience (4). In turn, Rissanen et al. (36) demonstrated that the traditional sauna poses a strenuous load and should not be recommended 24 h before the next training session. Hormonal responses to exercise loadings did not change after sauna. Physical activity is associated with oxidative stress, and as a result of regular physical training, the body adapts to the changes in the oxidant-antioxidant balance, which increases antioxidant capacity (37, 38). Sutkowy et al. (13) found that a high-intensity 30 min aerobic cycle ergometer test (approx. 80% of HRmax), followed by ice-water immersion (3°C, 5 min) or recovery at room temperature had no considerable effect on the serum activities of selected lysosomal enzymes and 1-antitrypsin in young (aged 18) amateur football players. These findings suggest that the stability of lysosomal membranes in tissues (in particular in striated muscles) was not significantly affected by exercise followed by immersion or recovery. Exercise/recovery decreased hydrogen peroxide concentration in erythrocytes, whereas exercise/immersion exerted the opposite effect. Pawłowska et al. (15) reported that cold water immersion appeared to be more effective than sauna in reducing the inflammatory response after exercise.

Sauna bathing is generally considered safe, and it is usually well tolerated by people of all ages, from children to seniors (19). However, inadequate sauna use can pose a health risk (39). Hot air sauna burns (HASB) are not encountered frequently, but they can be potentially lethal, with simultaneous rhabdomyolysis. Burns are sustained when immobile sauna bathers are exposed to hot air for long periods of time. The parts of the body that are directly exposed to hot air are most likely to be affected. As a result, the heat effects accumulate on the most exposed body parts and cause the so called “Apex Burns.” Exposure to hot air can lead to complex injuries, where full-thickness skin damage is accompanied by deeper tissue damage (40, 41). In a study by Ghods et al. (39), 16 sauna users sustained burns between 1999 and 2006, and the percentage of burned body surface was determined at 5–25%. In a Finnish study of sauna-related burns, 7% of all burns were caused by hot air (11 out of 154 patients) (42).

However, bodily responses to extremely high temperatures in the sauna have been less well researched. Sporadic sauna users may be unaware of safe temperature limits and are often exposed to excessive thermal stress in the sauna. Therefore, the aim of this study was to determine the effect of 20 min sauna sessions with temperatures of 80°C and 120°C on the physiological and psychological characteristics of young women who sporadically use the sauna. The results of this study provide valuable inputs for managing wellness and SPA centers.

## 2 Materials and methods

### 2.1 Statement

The research methodology used in this study has been applied in previous studies investigating the effect of thermal stress on the physiological characteristics of young adults, conducted by the authors, whose results have already been published (references 56 to 65). According to the reviewers, the adopted methods are reliable and the obtained results are objective. Therefore, the research methodology and the presentation of results in this study are similar to those in previous articles by the authors who assume full responsibility for the content and structure of this manuscript.

### 2.2 Participant selection

The study was conducted on 22 full-time university female students aged 18–27 years (22.26 ± 3.15) who volunteered for the research. Potential participants were informed about the purpose of the study during obligatory physiology classes at the University of Warmia and Mazury in Olsztyn (UWM). Those of them who agreed to participate in the experiment (28 women) received e-mail notifications and text messages informing them whether they met the inclusion criteria, and inviting them to the final stage of recruitment. Menstruating females were excluded from the study. Twenty-two female university students meeting the inclusion criteria were selected from a group of 26 volunteers and recruited for the study. All participants were examined by a physician, and they declared, in the physician’s presence, that they did not take any medications or nutritional supplements, were in good health, and had no history of blood diseases or diseases that could affect biochemical and biomechanical parameters. None of the evaluated participants had respiratory or circulatory ailments. In addition, the participants were closely supervised by a paramedic and a cardiologist during the entire study.

#### 2.2.1 Analysis I – anthropometric and body composition parameters

The descriptive statistics of anthropometric and body composition parameters are shown in Table 1. No significant deviations from normal distribution were found in any of the analyzed parameters. An analysis of the mean (898.0 MET) and minimum-maximum energy expenditure values (680-1,150 MET) demonstrated that the participants were characterized by moderate PA levels. The mean BMI value was within the norm (21.34 kg/m^2^), and WHR (0.84) was not indicative of gynoid or android obesity. However, total BFM was excessive relative to FFM, and the participants should lose 1 kg of fat tissue to achieve the target weight (FFM control – 2.1 kg).

**Table 1:** Descriptive statistics of the studied anthropometric and body composition parameters.

Parameter	Mean	SD	Min–Max	As
Age [years]	22.26	3.15	18–27	−0.14
PA [METs/min/week]	898.0	135.5	680–1,150	0.13
Body height [cm]	166.41	5.07	158–176	0.43
Body mass [kg]	59.10	8.43	48.1–84.8	1.32
Weight control [kg]	1.01	7.18	−19.3–7.9	−0.76
BMI (Body Mass Index) [kg/m^2^]	21.34	2.80	16.6–29.3	0.97
WHR (Waist-Hip Ratio)	0.84	0.04	0.78–0.96	1.02
TBW (Total Body Water) [L]	32.46	2.83	27.7–39.3	0.62
Proteins [kg]	8.70	0.78	7.2–10.5	0.35
Minerals [kg]	3.18	0.31	2.8–3.9	1.04
SMM (Skeletal Muscle Mass) [kg]	24.31	2.33	20.1–29.6	0.40
PBF (Percent Body Fat) [%]	24.38	5.64	13.2–40.5	0.93
BFM (Body Fat Mass) [kg]	14.76	5.74	6.4–34.4	1.13
BFM control [kg]	−1.04	5.47	−19.3–7.9	−1.18
FFM (Fat Free Mass) [kg]	44.34	3.90	37.8–53.7	0.62
FFM control [kg]	2.05	2.83	0.0–8.6	1.12
VFL (Visceral Fat Level) [kg]	5.68	2.78	2–16	1.63
Target weight [kg]	60.11	4.24	53.7–69.8	0.62

### 2.3 Ethical approval

The study was conducted upon the prior consent of the Ethics Committee of the UWM in Olsztyn (No. 10/2020), Poland. The participants were volunteers who signed an informed consent statement.

### 2.4 Instruments and procedures

#### 2.4.1 Assessment of physical activity levels

Physical activity (PA) levels (quantitative analysis) were assessed using the Polish short version of the standardized and validated International Physical Activity Questionnaire (IPAQ) (43). The IPAQ was used only to select a homogenous sample of female students, and the results were presented only in terms of metabolic equivalent of task (MET) units indicative of the participants’ PA levels. Before the experiment, the participants declared the average weekly number of minutes dedicated to PA (minimum of 10 min). The energy expenditure associated with weekly PA levels was expressed as METs-min/week (44). The MET is the ratio of the work metabolic rate to the resting metabolic rate, and 1 MET denotes the amount of oxygen consumed in 1 min, which is estimated at 3.5 mL/kg/min. Based on the declared frequency, intensity and duration of PA, the students were classified into groups characterized by low (<600 METs-min/week), moderate (600 to 1,500 METs per week), and high (≥1,500 METs-min/week) levels of activity. Only the females whose PA levels were classified as moderate and who had sporadically visited a sauna (1–3 times in their lifetime) were chosen for the study. A previous study of university students demonstrated that PA levels can significantly affect physiological parameters during sauna use (45), and in the present study, the participants’ PA levels were assessed to obtain a relatively homogeneous sample.

#### 2.4.2 Anthropometric and body composition parameters

Body height was measured to the nearest 1 mm with an InLab stadiometer (InBody Poland, Maniac Gym A.B.H Leszczyńscy, Białystok, Poland) equipped with ultrasound sensors that measure the distance from the head to the floor in 1 s. Anthropometric parameters, including body mass, body mass index (BMI), body surface area (BSA), and the waist-hip ratio (WHR), were measured directly before the first sauna session by bioelectrical impedance (46) with the InBody 270 body composition analyzer (InBody Poland, Maniac Gym A.B.H Leszczyńscy, Białystok, Poland). The same equipment was used to determine body composition parameters, including total body water (TBW), protein and mineral content, body fat mass (BFM), fat-free mass (FFM), skeletal muscle mass (SMM), percent body fat (PBF), InBody score, target weight, visceral fat level (VFL), basal metabolic rate (BMR), and degree of obesity. It was assumed that a seven-day interval between sauna sessions would not induce significant changes in the participants’ anthropometric and body composition parameters. To determine the loss of bodily fluids, body mass was measured directly before and after each sauna session with the InBody 270 analyzer.

#### 2.4.3 Physiological parameters

Before the experiment, all participants were familiarized with sauna rules. They were asked to drink minimum 1 L of water on the day of the trial and 0.5 L of water 2 h before the sauna session. The participants did not consume any foods or fluids until after the final body measurements at the end of the experiment. In order to eliminate the effect of diurnal variation on the experimental results, all participants visited a dry sauna in the same location, always between 8:00 and 10:00 a.m. (22). Every participant attended two sauna sessions (session I: temperature – 80°C; relative humidity – 14–16%; session II: temperature – 120°C, humidity – 12–14%) of 20 min each and remained in a sitting position during each session. The first and the second sauna session were separated by a period of 1 week. After each session, female students recovered in a neutral room (temperature of 18°C and relative humidity 40–50%) in a sitting position. Each recovery session lasted 6 min, during which the participants remained in a cold paddling pool (water temperature: 10–11°C) for 1 min (body immersion up to the neck). The entire experiment lasted 26 min (20 min of heating +6 min of cooling) for each session. Air temperature and humidity inside the sauna cabin and the neutral room, and water temperature in the paddling pool were measured with a Voltcraft BL-20 TRH + FM-200 hygrometer (Voltcraft Engineers Private Limited, Varanasi, India) and confirmed with a Stalgast 620,711 laser thermometer (Stalgast sp. z o.o., Radom, Poland).

Since the temperature in the sauna was high, during each sauna session, physiological parameters, including HR (minimum, average, peak), energy expenditure (kcal), and physical effort (based on a range of HR measurements), were assessed indirectly with heart rate monitors (Polar V800, Polar Electro, Kempele, Finland) that are widely used in studies of the type (47–49). Heart rate monitors were placed on the participants’ wrists, and HR sensors were attached to their chests. Before sauna bathing, each HR monitor was programmed for female sex, year of birth, body mass, and body height. During the first (80°C) and the second session (120°C), heart rate monitors were placed on the left wrist and were covered with the right hand to prevent burns. Body temperature cannot exceed 40°C (or can exceed 40°C insignificantly) to avoid hyperthermia. To prevent burns and overheating of heart monitors, sauna users were asked to place the other hand on top of the monitor. Based on HR values, the intensity of physical effort (low to high) was calculated in the Polar Flor application to determine the duration of each effort range during the sauna session. Physiological parameters (blood pressure, RH, and temperature) were also measured before sauna entry and immediately after the 20 min session during the 6 min cooling period. Blood pressure (SBP and DBP) and HR were measured with an automatic digital blood pressure monitor (Omron M6 Comfort, Tokyo, Japan), and forehead temperature was measured with the Buerer FT90 clinical contactless thermometer (Buerer Medical GmbH, Söflinger, Germany).

#### 2.4.4 Wellbeing assessment

The Profile of Mood States (POMS) questionnaire was used to evaluate the participants’ wellbeing. The questionnaire measures six different mood states, namely tension, depression, anger, fatigue, confusion, and vigor, and it contains 65 adjectives referring to those mood states. The “tension” subscale describes perceived muscle tension and generalized discomfort. The “depression” subscale describes the feelings of sadness, inadequacy, and worthlessness. “Anger” is characterized by irritability and hostility toward others. “Vigor” denotes emotional action readiness and invigoration. “Fatigue” is described as a feeling of exhaustion and a lack of energy. “Confusion” is defined as a confused state of mind and a feeling of ineffectiveness. The intensity of the mood states experienced at a given moment is evaluated on a five-point scale: 0 – definitely not, 1 – rather not, 2 – hard to say, 3 – rather yes, 4 – definitely yes. The POMS questionnaire has an overall detection rate of 80%, sensitivity of 55%, and specificity of 84% (50), and it is a valid and reliable tool for measuring negative emotional states such as psychological distress, and positive feelings such as vigor (51), including in conjunction with PA (45, 52).

#### 2.4.5 Assessment of heat illness symptoms

The term heat illness (HI) refers to incapacitating conditions that are directly associated with a rise in body temperature, and it can include heat stroke (HS), as well as milder disorders such as heat exhaustion (HE), heat syncope, heat cramps, and heat rash (53).

The health risks associated with exposure to high temperature were evaluated by analyzing which symptoms characteristic of HE and HS occurred during both sauna sessions (54). An anonymous questionnaire survey was conducted to obtain the relevant information from the participants. All symptoms typical of HE and SE were listed in the questionnaire:

HE: Heavy sweating; cold, pale, and clammy skin; fast weak HR; nausea or vomiting; muscle cramps; tiredness or weakness; dizziness; headache; fainting (syncope).

HS: High body temperature (103°F or higher); hot, red, dry, or damp skin; fast strong HR; headache; dizziness; nausea; confusion; loss of consciousness (syncope). Participants who had experienced a given symptom placed an “x” in the corresponding column. Heart rate was measured with Suunto Ambit3 Peak Sapphire HR monitors (described in the *Physiological parameters* subsection).

### 2.5 Statistical analysis

Basic descriptive statistics (mean, SD, and variation) were calculated for each parameter, and the data were checked for the normality of distribution (Shapiro–Wilk test and the asymmetry coefficient). All analyzed parameters were normally distributed, and the Student’s *t*-test for dependent samples was used to estimate the significance of differences between the mean values of the tested parameters before and after sauna. In addition, Cohen’s *d* was used to assess the effect size of these differences. Cohen’s *d* was interpreted as follows: trivial (<0.2), small (0.21–0.6), moderate (0.61–1.2), large (1.21–1.99), and very large (>2.0) differences. The calculations were performed at a significance level of α = 0.05, in the Statistica ver. 13 program. The values determined in three women who had fainted during a 20 min sauna session with a temperature of 120°C and had to leave the sauna room before the end of the session (after 18:37 to 19:01 min from the beginning of the session) were also considered in the statistical analysis because the time remaining until the end of the 20 min session had no significant impact on the obtained HR values.

The presence of significant correlations between HE and HS variables and syncope during the second sauna session was determined with the use of classification and regression trees (CRT) and the cross-validation technique. A model was developed only for the temperature of 120°C because syncope had not occurred during the first sauna session with the temperature of 80°C. The developed model involved 11 independent variables. Nausea, heavy sweating, fast weak pulse, high body temperature, and confusion induced the greatest differences in the reported outcomes (all of these parameters received a score of 2 points in the survey). The tree analysis revealed that the model had high predictive strength (95.5%) with a specificity of 100% and sensitivity of 66.7% (Table 2).

**Table 2:** Quality analysis of the classification and regression tree.

Quality measurement	Value
Accuracy	95.5%
Sensitivity	66.7%
Specificity	100%

## 3 Results

### 3.1 Analysis I – anthropometric and body composition parameters

The descriptive statistics of anthropometric and body composition parameters are shown in Table 2. No significant deviations from normal distribution were found in any of the analyzed parameters. An analysis of the mean (898.0 MET) and minimum-maximum energy expenditure values (680–1,150 MET) demonstrated that the participants were characterized by moderate PA levels. The mean BMI value was within the norm (21.34 kg/m^2^), and WHR (0.84) was not indicative of gynoid or android obesity. However, total BFM was excessive relative to FFM, and the participants should lose 1 kg of fat tissue to achieve the target weight (FFM control – 2.1 kg).

### 3.2 Analysis II – changes in physiological parameters

Changes in physiological parameters (SBP, DBP, HR, forehead temperature) induced by thermal stress in the sauna are presented in Table 3. The mean values of SBP and DBP decreased significantly during 20 min sauna sessions with a temperature of 80°C (*p* < 0.001 and *p* = 0.015, respectively) and 120°C (excluding SBP, *p* < 0.001). No significant differences in the values of BP (SBP and DBP), forehead temperature, or body mass were noted directly before and after the first (80°C) and second (120°C) sauna session.

**Table 3:** Descriptive statistics of the parameters evaluated before and after sauna with a temperature of 80°C and 120°C.

Parameter	Temperature [^o^C]	Before sauna			After sauna		Differences			
		Mean	SD	Min–Max	Mean	SD	Min–Max	*t*	*p*	Cohen’s *d*
SBP [mmHg]	80	110.45	9.11	95–127	104.77	8.80	82–120	−4.87	<0.001	0.64
	120	108.41	7.40	95–123	107.41	7.87	93–122	−0.53	ns	0.13
		ns			ns					
DBP [mmHg]	80	72.00	6.65	59–91	69.05	6.83	52–80	−2.65	0.015	0.44
	120	72.55	4.57	61–81	67.14	7.08	51–78	−3.98	<0.001	0.91
		ns			ns					
HR [bpm]	80	79.27	9.23	57–100	114.23	9.54	97–127	12.97	<0.001	3.72
	120	82.68	11.09	57–99	120.95	16.46	80–155	9.26	<0.001	2.73
		ns			ns					
Forehead temperature [^o^C]	80	36.66	0.25	35.9–37.1	37.91	0.45	37.1–38.7	11.01	<0,001	3.43
	120	36.84	0.42	36.0–37.7	38.24	0.78	37.1–40.2	7.87	<0,001	2.23
		ns			ns					
Body mass [kg]	80	59.41	8.43	48.1–84.8	59.27	8.44	48.1–84.7	−9.92	<0.001	0.02
	120	58.12	7.46	49.0–84.8	57.82	7.45	48.8–84.4	−13.16	<0.001	0.04
		ns			ns					

ns, no significant difference.

The mean values of HR (min, avg., and peak) and energy expenditure were significantly higher (*p* < 0.001) during a 20 min stay in the sauna with a temperature of 120°C than 80°C (Table 4). During the first and second sauna session, the participants remained in the moderate effort range for the longest time (532.5 s and 354.1 s, respectively), and no significant differences were observed between sessions (*p* > 0.05). However, the duration of the low effort range in the first session (421.2 s) and the difficult effort range in the second session (354.1 s) was also relatively long. In addition, the maximum effort range was exceeded for 19.2 s during the second session, whereas no such observations were made during the first session.

**Table 4:** Comparison of the parameters evaluated during sauna with a temperature of 80°C and 120°C.

Parameter	80^o^C			120^o^C			Differences		
	Mean	SD	Min–Max	Mean	SD	Min-Max	*t*	*p*	Cohen’s *d*
HR min.	77.45	9.59	56–95	88.95	10.05	69–113	3.69	<0.001	1.17
HR avg	102.73	11.24	70–117	120.82	9.14	104–139	5.71	<0.001	1.77
HR peak	127.73	10.98	100–145	145.45	9.05	127–162	5.57	<0.001	1.82
Energy expenditure	118.55	24.79	80–169	185.95	25.18	158–245	8.40	<0.001	2.70
Duration of HR effort ranges (physical intensity) [s]									
Very low, 104 – 114 [bpm]	120.73	154.91	0–540	18.41	46.94	0–199	−2.86	0.009	0.89
Low, 114 – 133 [bpm].	421.23	261.84	37–937	183.86	241.48	0–735	−3.13	0.005	0.84
Moderate, 133 – 152 [bpm]	532.50	259.87	83–1148	446.32	227.32	34–924	−1.31	ns	0.35
High, 152 – 172 [bpm]	112.27	111.50	0–356	354.09	221.40	8–769	4.80	<0.001	1.38
Very high, 172 – 190 [bpm]	13.27	38.78	0–172	167.18	226.11	0–653	3.05	0.006	0.95
Maximal > 190 [bpm]	0.00	0.00	0–0	19.23	62.76	0–285	1.43	ns	0.43

An analysis of the results presented in Table 5 indicates that tension, depression, anger, fatigue, and confusion decreased significantly (*p* < 0.001 for all POMS variables), whereas vigor increased after the sauna session with a temperature of 80°C. The effect size was high in all cases. In turn, exposure to a temperature of 120°C induced a significant increase (*p* < 0.001 for all POMS variables) in tension, depression, fatigue, and confusion, and a decrease in vigor. Tension was significantly higher before the second sauna session (120°C, *p* < 0.014), whereas depression and anger were significantly higher before the first sauna session (80°C, *p* = 0.001–0.012) than the second session. Tension, depression, anger, fatigue, and confusion were significantly higher (*p* < 0.001) after the second (120°C) than the first (80°C) session. Vigor was the only exception, and it was higher after the first than the second session (*p* < 0.001) (Table 5).

**Table 5:** Comparison of POMS parameters before and after sauna with a temperature of 80°C and 120°C.

POMS variables	Temperature [^o^C]	Before sauna			After sauna			Differences		
		Mean	SD	Min–Max	Mean	SD	Min–Max	*t*	*p*	Cohen’s *d*
Tension	80	10.82	3.32	5–18	5.95	3.12	3–13	−9.15	<0.001	1.95
	120	13.05	3.20	7–22	20.50	2.46	17–28	9.48	<0.001	2.02
		*p*=0.014; *d* Cohen = 0.68			*p*<0.001; *d* Cohen = 5.18					
Depression	80	10.41	4.39	0–17	2.73	1.75	0–7	−8.46	<0.001	1.80
	120	8.50	2.39	4–12	12.23	3.10	7–20	4.87	<0.001	1.04
		*p*=0.012; *d* Cohen = 0.54			*p*<0.001; *d* Cohen = 3.77					
Anger	80	6.09	2.62	3–13	0.59	0.80	0–3	−9.25	<0.001	1.97
	120	3.68	1.64	1–9	3.18	1.74	0–6	−1.11	ns	0.24
		*p*=0.001; *d* Cohen = 1.10			*p*<0.001; *d* Cohen = 1.91					
Fatigue	80	8.64	5.41	1–18	2.41	1.40	0–6	−5.40	<0.001	1.15
	120	7.86	2.68	3–13	13.27	2.69	8–18	6.45	<0.001	1.37
		ns			*p*<0.001; *d* Cohen = 5.06					
Confusion	80	4.23	2.35	1–8	1.86	1.32	0–5	−5.21	<0.001	1.11
	120	4.05	1.65	0–7	13.50	2.56	10–18	15.52	<0.001	3.31
		ns			*p*<0.001; *d* Cohen = 5.71					
Vigor	80	16.59	2.92	11–21	22.41	1.89	20–26	9.61	<0.001	2.05
	120	15.41	2.61	12–23	2.36	1.18	0–5	−23.29	<0.001	4.97
		ns			*p*<0.001; *d* Cohen = 12.73					

**Figure 1:**
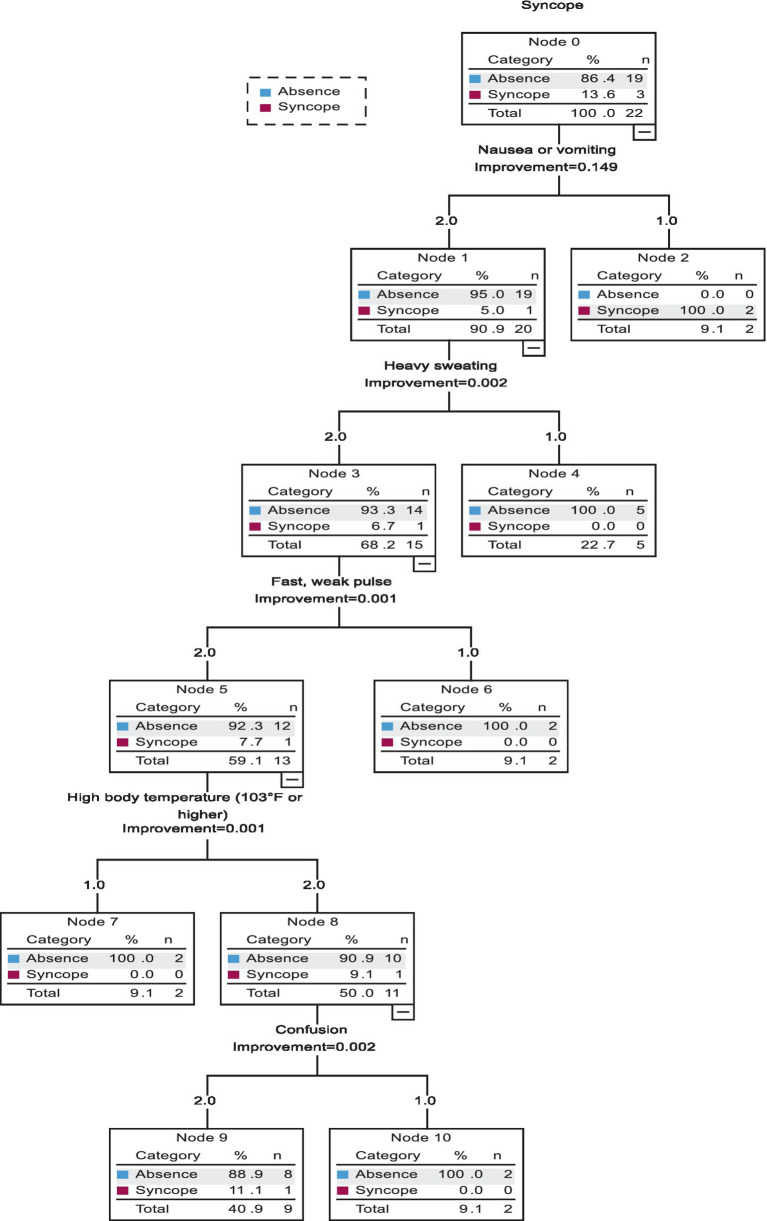
Classification and regression tree (CRT) for model 1.

The above analysis (Figure 1) revealed that vomiting was the best predictor of syncope. Vomiting occurred directly before syncope in two out of the three women who lost consciousness. The third woman did not vomit, but she reported heavy sweating, fast weak HR, high body temperature, and confusion. All of the above symptoms are important, but non-specific because they also occurred in eight other women who did not lose consciousness. These observations indicate that other, more specific factors that contribute to syncope should be included in the analysis.

**Figure 2:**
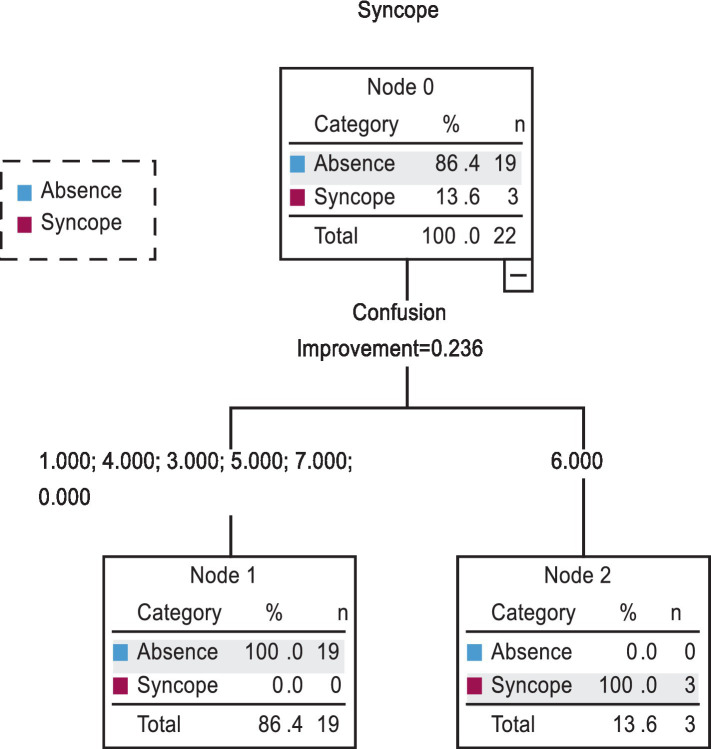
Classification and regression tree (CRT) for model 2.

In the second CRT model (Figure 2), only POMS variables (tension, depression, anger, fatigue, and confusion) were used as predictors of syncope. However, confusion at level 6 was the only significant predictor of syncope that was experienced by 100% of the tested subjects before sauna. The specificity of the CRT was determined at 100%. Confusion at level 7 was reported by only 1 participant before sauna, and it did not lead to syncope. Therefore, confusion at level 6 and higher should be regarded as a contraindication for sauna bathing at a temperature of 120°C.

## 4 Discussion

Sauna bathing is an increasingly popular recreational activity around the world, which is why research into safe sauna use has important implications for bathers’ health. The aim of this study was to determine the effect of 20 min sauna sessions with a temperature of 80°C and 120°C on the physiological and psychological parameters of young women who were sporadic sauna users. The observed changes in the analyzed parameters were influenced by temperature inside the sauna chamber. Prolonged exposure to very high temperatures can produce adverse side effects that are potentially dangerous to human health. Three out of the 22 women attending a 20 min sauna session at a temperature of 120°C lost consciousness and had to leave the sauna chamber before the end of the session (after around 18:30 min from the beginning of the session). Shin et al. (55) described a case of a 70-year-old woman who slept for 30 min during a sauna session. Her right medial thigh was covered with a fully wet towel. The patient sustained a second-degree burn with multiple bullas in the right medial thigh region. A physical examination revealed erythema, heating sensation, and swelling around the bullas. These observations indicate that sauna should be used with caution and that all bathers should strictly adhere to sauna safety rules.

### 4.1 Changes in physiological parameters

An analysis of changes in BP values revealed a significant decrease in SBP after the first sauna session (80°C) and a significant decrease in DBP after both sauna sessions (80°C and 120°C). A significant decrease in SBP and DBP was also observed in women subjected to repeated alternative thermal stress during four sauna sessions (temperature: 90–91°C, humidity: 14–16%) of 12 min each, interspersed with four recovery sessions of 6 min each during which the participants remained in cold water (9–10°C) (22). In turn, a 30 min sauna session (temperature: 73°C, humidity: 10–12%) involving a 2 min shower break led to a decrease in SBP (from 135.8 to 128.9 mmHg) and DBP (from 79.6 to 72.5 mmHg) in women with at least one cardiovascular risk factor (30).

After both sauna sessions, HR values increased significantly and remained within the low effort range. However, the HR values registered after the second sauna session (120°C) were higher by 6.7 bpm. In another study, the HR values of middle-aged women (52 years) increased from 65 to 75 bpm. However, it should be noted that the mean and maximal values of HR tend to decrease with age (56), which is why various formulas are used to estimate HR_max_ [e.g., HR_max_ = 220 – age (57), or HR_max_ = 205−0.5 • age (58)] and select exercise intensity that is appropriate for the subject’s age and physiological capacity (59). Despite individual differences, the procedure of calculating maximal HR values can be also useful for determining the optimal temperature during sauna. High temperature and prolonged sauna bathing can exert a significant load on the cardiovascular system in both seniors (60) and athletes. As a result, instead of promoting relaxation and delivering health benefits, sauna can exacerbate fatigue (4). According to specialists, for persons with cardiovascular problems (such as chronic congestive heart failure), the temperature inside the sauna chamber should be lowered to even 60°C, and each session should be followed by a recovery period (up to 30 min) with oral hydration (61–63). In most cases, the optimal temperature in a Finnish sauna ranges from 80°C to 90°C at face level (4, 20). A steam sauna is yet another type of sauna treatment with a temperature of only 45–50°C, but nearly 100% relative humidity (64). Infrared sauna, which does not involve steam and is characterized by low air temperature (40–65°C), has become popular in recent years, in particular in the clinical setting (3). Therefore, the temperature applied in the Finnish sauna (120°C) in the present study should be regarded as extremely high, especially since the participants were sporadic sauna users. Equally high or even higher temperatures are sometimes applied in sauna practice (64). A study of well-trained male cyclists who regularly used the sauna revealed that exposure to extreme heat stress in a sauna can induce changes in HR and cardiac autonomic modulations (65). In irregular sauna users, a HR increase to around 120 bpm is a desirable adaptive response, whereas HR above 140 bpm can be unfavorable because it is accompanied by a rise in cardiac work and shortening of the diastole (64, 66). Cardiovascular responses to high-temperature (80°C) sauna baths include tachycardia, systolic hypertension, and increased cardiac workload, which are mediated by peripheral vasodilatation and stimulation of the sympathetic nervous system (63).

An analysis of the duration of each HR effort range revealed that the cardiovascular responses of the studied women were more dynamic during the second session (120°C) and even reached the maximal effort range (>190 bpm for approx. 20 s). Similar observations were made by Ketelhuts (67) who examined 19 healthy volunteers (36.8% women) before and after a 25 min sauna session (temperature: 93°C, humidity: 13–20%). The cited authors reported a continuous increase in BP and HR during the sauna session, followed by a sustained decrease in the values of the remaining parameters after heat exposure, which suggests that sauna bathing has a beneficial effect on the cardiovascular system. Furthermore, the results indicate that acute heat exposure in the sauna exerts a burden that is comparable to moderate physical exercise.

Exposure to 80°C and 120°C led to a significant increase in forehead temperature which approximated the values reported in 52-year-old women (38°C) directly after the second 15 min sauna session (temperature: 73°C, humidity: 10–12%) (17). During a sauna bath, the body absorbs more heat from the surrounding environment than it can release and, in consequence, skin temperature and body temperature may reach 37.6–40°C (22, 64, 68).

After each sauna session (80°C and 120°C), a significant decrease (0.14 kg and 0.3 kg, respectively) was observed in the participants’ body mass. This result indicates that sweating intensified at higher temperature to maintain a stable body temperature. Research conducted on female university students demonstrated that the duration and temperature of sauna treatment influence sweating. Repeated alternative thermal stress (temperature: 90–91°C, humidity: 14–16%) decreased body mass by 0.3 kg on average (22). In a study of 326 female university students, two dry sauna sessions (temperature: 90°C, humidity: 35%) of 10 min each with a 5 min break involving a 30 s cooling period in a paddling pool (water temperature: 10°C) decreased body mass by 0.5 kg (69). In young women, sweating increased with a rise in temperature and humidity or prolonged stay in the sauna (70), and it was also affected by the duration of the break and the temperature of cooling water (16, 22). A study performed on male subjects also revealed that body mass loss (mainly the loss of bodily fluids) can be exacerbated by excessive body fat (48, 49), as well as PA level and frequency of sauna use (45). These observations have important practical implications, especially for individuals who take long sauna baths and regard sauna as an effective therapy for burning fat tissue (22). However, sauna-induced weight loss stirs considerable controversy. Prolonged and intensive body cooling in cold water slows down the metabolism and, consequently, enables users to significantly extend their stay in the sauna. Finnish sauna treatments should involve cooling-off periods and rehydration with oral fluids during and/or after each session to increase the effectiveness of rehydration and body cooling (71). Cold water and ice slurry drinks are body-cooling methods that can be easily administered to sauna users and individuals who exercise in heat. Cold fluid ingestion stimulates thermoreceptors in the abdomen that appear to reduce sweat production independently of core and skin temperature. Therefore, cold fluid ingestion during sauna can improve the users’ thermal comfort and perceptual responses, and it can induce transient changes in sweating (72). A study of athletes demonstrated that drinking cold water can considerably modulate and delay an increase in the core body temperature of euhydrated subjects who exercise in a moderate climate (73).

### 4.2 Changes in psychological parameters

Most wellbeing components, including tension, depression, anger, fatigue, and confusion decreased significantly, whereas vigor increased after a 20 min sauna session with a temperature of 80°C. A sauna session with a temperature of 120°C induced the opposite effects: tension, depression, and fatigue increased significantly, whereas vigor decreased. These results suggest that the wellbeing of women who sporadically use the sauna improves under exposure to a temperature of 80°C, but deteriorates under exposure to a temperature of 120°C. A study conducted on 550 female university students demonstrated that a 20 min sauna session (temperature: 90°C, humidity: 14–16%) exerted a positive influence on their mental wellbeing (34). In turn, Kanji et al. (35) found that regular sauna bathing (three 20 min sessions per week, no temperature or humidity data) is a simple, self-directed treatment that effectively reduces the severity of chronic tension-type headache. Similar results were reported by Masuda et al. (74) who observed that multidisciplinary treatment combined with repeated far-infrared ray dry sauna therapy (temperature: 60°C, duration: 15 min) could be a promising method for chronic pain management.

According to the Global Sauna Survey, sauna bathers, particularly those from Finland, Australia, and the United States, visited sauna mainly for relaxation and stress reduction and reported health benefits, especially improved mental wellbeing and sleep, with relatively few adverse effects. Interestingly, unlike in the present study, the evaluated subjects used the sauna 4–12 times each month (75). Most respondents used traditional Finnish-style saunas within a temperature range of 60–90°C, which was much lower than the temperature of the second sauna session in the present study. In the present study, syncope occurred in women who experienced confusion at level 6 on a 7-point scale. In two cases, vomiting directly preceded loss of consciousness. This information has practical implications for therapists and persons conducting physical activity programs that involve sauna. Therapists should pay special attention to symptoms characteristic of heat illness, including those that did not occur in this experiment, because responses to thermal stress can vary across individuals, as confirmed by the current study.

### 4.3 Adaptation to thermal stress

The present study makes a reference to research examining the optimal temperature during thermotherapy (76–79). The hypothalamus plays a key role in autonomic thermoregulation in humans (80). Individuals with heat intolerance experience a more rapid and more pronounced increase in body temperature and higher physiological strain than heat-tolerant individuals. Heat acclimation is achieved through the thermoregulation mechanism. Heat acclimation implies that an organism is able to reach and maintain a thermal equilibrium at high temperatures (81). For this effect to be achieved, sauna has to be used regularly, whereas the women examined in this experiment were sporadic sauna users. For example, it was found that repeated heat exposure over a period of 4 days to 2 weeks induced cardiovascular and thermoregulatory adaptations in domestic animals, thus decreasing physiological strain and increasing heat tolerance (82). Mee et al. (83) reported that sauna exposure immediately prior to controlled hyperthermia and heat acclimation reduced thermoregulatory, cardiovascular, and perceptual strain when running a heat tolerance test. Classic physiological adaptations after heat acclimation include an increase in sweat loss and a decrease in core temperature, skin temperature, and HR (84). In the current study, thermoregulatory sweating was less profuse, probably because the examined women were sporadic sauna users. This observation could explain why the registered physiological parameters (HR and BP), unlike psychological parameters, were more ambiguous markers of hyperthermia at a temperature of 120°C.

The findings from prospective studies suggest that women are at increased risk of heat intolerance. Physical factors such as the BMI, BSA, and PBF considerably affect body temperature responses to heat, equally in both sexes (85). However, in females, cyclic variation in estrogen and progesterone levels is associated with thermoregulatory changes: the core body temperature increases, and there is a rightward shift in the temperature threshold for the onset of vasodilation in the luteal phase, which is also observed in women who take many hormonal contraceptives (86).

### 4.4 Strengths and limitations

The main strength of the study is that it is the only research to have examined sauna bathing at extremely high temperatures to date. The relatively small, but carefully selected sample of young women is a possible limitation of the study. In the future, the results should be validated on a larger sample of both female and male participants to determine gender-related differences in adaptation to thermal stress.

## 5 Conclusion

The study demonstrated that 20 min sauna sessions at a temperature of 80°C and 120°C induced significant changes in the bathers’ physiological and psychological characteristics. Both temperatures led to a significant decrease in SBP (excluding at 120°C), DBP, and body mass, and a significant increase in HR and forehead temperature. A 20 min sauna session at a temperature of 80°C increased vigor and decreased tension, depression, anger, fatigue, and confusion. A sauna session at a temperature of 120°C exerted the opposite effects by inducing negative changes in the participants’ mental wellbeing. A temperature of 120°C in a Finnish sauna can induce symptoms characteristic of heat exhaustion and heat stress, including syncope, nausea, heavy sweating, fast weak pulse, high body temperature, and confusion. Confusion and vomiting were the main predictors of syncope in three women. Therefore, sauna bathing at a temperature of 80°C is recommended for young women who are sporadic sauna users, whereas 120°C is excessive and could lead to syncope. Syncope could be followed by loss of consciousness (87) with potentially serious health complications or even death. The present findings provide highly valuable inputs for managing wellness and SPA centers.

## Data availability statement

The datasets presented in this article are not readily available because the access to Excel data has been restricted by the Ethics Committee of the UWM in Olsztyn to protect the participants’ privacy. Researchers who meet the criteria for access to confidential data can submit a data request by email. Requests to access the datasets should be directed to podstawskirobert@gmail.com.

## Ethics statement

The studies involving humans were approved by Ethics Committee of the University of Warmia and Mazury in Olsztyn. The studies were conducted in accordance with the local legislation and institutional requirements. The participants provided their written informed consent to participate in this study. Written informed consent was obtained from the individual(s) for the publication of any potentially identifiable images or data included in this article.

## Author contributions

RP: Conceptualization, Data curation, Formal analysis, Funding acquisition, Investigation, Methodology, Writing – original draft, Writing – review & editing. KB: Formal analysis, Software, Supervision, Validation, Writing – review & editing. NJ: Formal analysis, Software, Supervision, Validation, Writing – original draft. JS: Data curation, Funding acquisition, Writing – original draft. BH: Data curation, Writing – original draft. EB: Formal analysis, Writing – review & editing. AP: Data curation, Writing – original draft.

## References

[1] Laukkanen T Lipponen J Kunutsor SK Zaccardi F Araújo CGS Mäkikallio TH. Recovery from sauna bathing favorably modulates cardiac autonomic nervous system. Complement Ther Med. (2019) 45:190–7. doi: 10.1016/j.ctim.2019.06.011, PMID: 31331560

[2] Pilch W Szygula Z Palka T Pilch P Cison T Wiecha S. Comparison of physiological reactions and physiological strain in healthy men under heat stress in dry and steam heat saunas. Biol Sport. (2014) 31:145–9. doi: 10.5604/20831862.1099045, PMID: 24899780 PMC4042662

[3] Imamura M Biro S Kihara T Yoshifuku S Takasaki K Otuji Y. Repeated thermal therapy improves impaired vascular endothelial function in patients with coronary risk factors. J Am Coll Cardiol. (2001) 38:1083–8. doi: 10.1016/s0735-1097(01)01467-x, PMID: 11583886

[4] Mero A Tornberg J Mäntykoski M Puurtinen R. Effects of far-infrared sauna bathing on recovery from strength and endurance training sessions in men. Springerplus. (2015) 4:321. doi: 10.1186/s40064-015-1093-5, PMID: 26180741 PMC4493260

[5] Yamasaki S Tokunou T Maeda T Horiuchi T. Hot spring bathing is associated with a lower prevalence of hypertension among Japanese older adults: a cross-sectional study in Beppu. Sci Rep. (2022) 12:19462. doi: 10.1038/s41598-022-24062-3, PMID: 36376349 PMC9661464

[6] He BJ Zhao D Dong X Xiong K Feng C Qi Q. Perception, physiological and psychological impacts, adaptive awareness and knowledge, and climate justice under urban heat: a study in extremely hot-humid Chongqing, China. Sustain Cities Soc. (2022) 79:103685. doi: 10.1016/j.scs.2022.103685

[7] Heinonen I Laukkanen JA. Effects of heat and cold on health, with special reference to Finnish sauna bathing. Am J Physiol Regul Integr Comp Physiol. (2018) 314:R629–38. doi: 10.1152/ajpregu.00115.2017, PMID: 29351426

[8] Yuan W He BJ Yang L Liu X Yan L. Heat-induced health impacts and the drivers: implications on accurate heat-health plans and guidelines. Environ Sci Pollut Res Int. (2022) 29:88193–212. doi: 10.1007/s11356-022-21839-x, PMID: 35829877

[9] Brunt VE Howard MJ Francisco MA Ely BR Minson CT. Passive heat therapy improves endothelial function, arterial stiffness and blood pressure in sedentary humans. J Physiol. (2016) 594:5329–42. doi: 10.1113/JP272453, PMID: 27270841 PMC5023696

[10] Didehdar D Sobhani S. The effect of cold-water immersion on physical performance. J Bodyw Mov Ther. (2019) 23:258–61. doi: 10.1016/j.jbmt.2018.05.001 31103105

[11] Greenwood A Gillette C. Effect of cold water immersion on metabolic rate in humans. Int J Kinesiol Sports Sci. (2017) 5:1–6. doi: 10.7575/aiac.ijkss.v.5n.2p.1

[12] Moore E Fuller JT Bellenger CR Saunders S Halson SL Broatch JR. Effects of cold-water immersion compared with other recovery modalities on athletic performance following acute strenuous exercise in physically active participants: a systematic review, meta-analysis, and meta-regression. Sports Med. (2023) 53:687–705. doi: 10.1007/s40279-022-01800-1, PMID: 36527593

[13] Sutkowy P Woźniak A Boraczyński T Boraczyński M Mila-Kierzenkowska C. The oxidant-antioxidant equilibrium, activities of selected lysosomal enzymes and activity of acute phase protein in peripheral blood of 18-year-old football players after aerobic cycle ergometer test combined with ice-water immersion or recovery at room temperature. Cryobiology. (2017) 74:126–31. doi: 10.1016/j.cryobiol.2016.11.004 27871846

[14] Xiao F Kabachkova AV Jiao L Zhao H Kapilevich LV. Effects of cold water immersion after exercise on fatigue recovery and exercise performance--meta analysis. Front Physiol. (2023) 14:1006512. doi: 10.3389/fphys.2023, PMID: 36744038 PMC9896520

[15] Pawłowska M Mila-Kierzenkowska C Boraczyński T Boraczyński M Szewczyk-Golec K Sutkowy P. The influence of ambient temperature changes on the indicators of inflammation and oxidative damage in blood after submaximal exercise. Antioxidants. (2022) 11:2445. doi: 10.3390/antiox11122445, PMID: 36552653 PMC9774713

[16] Podstawski R Borysławski K Clark CCT Laukkanen JA Gronek P. The effect of 16-minute thermal stress and 2-minute cold water immersion on the physiological parameters of young sedentary men. Montenegrin J Sports Sci Med. (2020) 9:57–65. doi: 10.26773/mjssm.200308

[17] Laukkanen T Kunutsor SK Zaccardi F Lee E Willeit P Khan H. Acute effects of sauna bathing on cardiovascular function. J Hum Hypertens. (2018) 32:129–38. doi: 10.1038/s41371-017-0008-z, PMID: 29269746

[18] The Finnish Sauna Society. Sauna and health. Available at: http://www.sauna.fi/64.html (Accessed May 5, 2011).

[19] Hannuksela ML Ellahham S. Benefits and risks of sauna bathing. Am J Med. (2001) 110:118–26. doi: 10.1016/S0002-9343(00)00671-9, PMID: 11165553

[20] Kukkonen-Harjula K Kauppinen K. Health effects and risk of sauna bathing. Int J Circumpol Heal. (2006) 65:195–205. 10.3402/ijch.v65i3.18102 16871826

[21] Kauppinen K. Sauna, shower, and ice water immersion. Physiological response to brief exposure to heat, cool, and cold. Part II. Circulation. Arctic Med Res. (1989) 48:64–74. 2736002

[22] Podstawski R Borysławski K Hinca B Finn K Dziełak A. Effect of repeated alternative thermal stress on the physiological and body composition characteristics of young women sporadically using sauna. Phys Act Rev. (2023) 11:49–59. doi: 10.16926/par.2023.11.07

[23] Gayda M Bosquet L Paillard F Garzon M Sosner P Juneau M. Effects of sauna alone versus postexercise sauna baths on short-term heart rate variability in patients with untreated hypertension. J Cardiopulm Rehabil Prev. (2012) 32:147–54. doi: 10.1097/HCR.0b013e318251ffeb, PMID: 22561416

[24] Radtke T Poerschke D Wilhelm M Trachsel LD Tschanz H Matter F. Acute effects of Finnish sauna and cold-water immersion on haemodynamic variables and autonomic nervous system activity in patients with heart failure. Eur J Prev Cardiol. (2016) 23:593–601. doi: 10.1177/2047487315594506, PMID: 26152773

[25] Tomiyama C Watanabe M Honma T Inada A Hayakawa T Ryufuku M. The effect of repetitive mild hyperthermia on body temperature, the autonomic nervous system, and innate and adaptive immunity. Biomed Res. (2015) 36:135–42. doi: 10.2220/biomedres, PMID: 25876664

[26] Zalewski P Zawadka-Kunikowska M Słomko J Szrajda J Klawe JJ Tafil-Klawe M. Cardiovascular and thermal response to dry-sauna exposure in healthy subjects. Physiol J. (2014) 2014:10. doi: 10.1155/2014/106049.106049

[27] Podstawski R Borysłąwski K Pomianowski A Krystkiewicz W Boraczyński T Mosler D. The effects of repeated thermal stress on the physiological parameters of young physically active men who regularly use the sauna: A multifactorial assessment. Int J Environ Res Public Health. (2021) 18:11503. doi: 10.3390/ijerph182111503 34770018 PMC8583525

[28] Strandberg TE Strandberg A Pitkälä K Benetos A. Sauna bathing, health, and quality of life among octogenarian men: the Helsinki businessmen study. Aging Clin Exp Res. (2018) 30:1053–7. doi: 10.1007/s40520-017-0855-z, PMID: 29188579

[29] Laukkanen T Khan H Zaccardi F Laukkanen JA. Association between sauna bathing and fatal cardiovascular and all-cause mortality events. JAMA Intern Med. (2015) 175:542–8. doi: 10.1001/jamainternmed.2014.8187, PMID: 25705824

[30] Laukkanen JA Laukkanen T Kunutsor SK. Cardiovascular and other health benefits of sauna bathing: a review of the evidence. Mayo Clin Proc. (2018) 93:1111–21. doi: 10.1016/j.mayocp.2018.04.008, PMID: 30077204

[31] Isomäki H. The sauna and rheumatic diseases. Ann Clin Res. (1988) 20:271–5. PMID: 3265298

[32] Nurmikko T Hietaharju A. Effect of exposure to sauna heat on neuropathicandr heumatoid pain. Pain. (1992) 49:43–51. doi: 10.1016/0304-3959(92)90186-F, PMID: 1375727

[33] Podstawski R Choszcz D Honkanen A Tuochino A Kolankowska E. Socio-economic factors and psycho-physical well-being as predictors of sauna use among male university students. S Afr J Res Sport Phys Educ Recreat. (2016) 38:163–76.

[34] Podstawski R Honkanen A Tuohino A Gizinska R. The influence of socioeconomic and psychological factors on the popularity of sauna treatment among female university students. Balt J Health Phys Act. (2015) 7:67–82. doi: 10.29359/BJHPA.07.2.06

[35] Kanji G Weatherall M Peter R Purdie G Page R. Efficacy of regular sauna bathing for chronic tension-type headache: a randomized controlled study. J Altern Complement Med. (2015) 21:103–9. doi: 10.1089/acm.2013.0466, PMID: 25636135

[36] Rissanen JA Häkkinen A Laukkanen J Kraemer WJ Häkkinen K. Acute neuromuscular and hormonal responses to different exercise loadings followed by a sauna. J Strength Cond Res. (2020) 34:313–322. doi: 10.1519/JSC.0000000000003371 31490429

[37] Peternelj TT Coombes JS. Antioxidant supplementation during exercise training: beneficial or detrimental? Sports Med. (2011) 41:1043–69. doi: 10.2165/11594400-000000000-00000 22060178

[38] Yavari A Javadi M Mirmiran P Bahadoran Z. Exercise-induced oxidative stress and dietary antioxidants. Asian J Sports Med. (2015) 6:e24898. doi: 10.5812/asjsm.24898, PMID: 25883776 PMC4393546

[39] Ghods M Corterier C Zindel K Kiene M Rudolf K Steendoi M. Hot air sauna burns. Burns. (2008) 34:122–4. doi: 10.1016/j.burns.2006.08.027, PMID: 17466460

[40] Koljonen V. Hot air sauna burns—review of their etiology and treatment. J Burn Care Res. (2009) 30:705–10. doi: 10.1097/BCR.0b013e3181abfa9f, PMID: 19506509

[41] Kluger N Laipio J Virolainen S Ranki A Koljonen V. A fatal case of hot air sauna burn in an elderly patient initially misdiagnosed as bullous Pemphigoid. Acta Derm Venereol. (2011) 91:732–3. doi: 10.2340/00015555-1140, PMID: 21727992

[42] Papp A. Sauna-related burns: a review of 154 cases treated in Kuopio University Hospital burn center 1994–2000. Burns. (2002) 28:57–9. doi: 10.1016/s0305-4179(01)00073-0, PMID: 11834331

[43] Biernat E Stupnicki R Gajewski AK. International physical activity questionnaire (IPAQ) – polish version. Phys Educ Sport. (2007) 51:47–54.

[44] Lee PH Macfarlane DJ Lam TH Stewart SM. Validity of the international physical activity questionnaire short form (IPAQ-SF): a systematic review. Int J Behav Nutr Phys Act. (2011) 8:115. doi: 10.1186/1479-5868-8-115, PMID: 22018588 PMC3214824

[45] Podstawski R Bielec G Borysławski K Alfoldi Z Marzec A. Changes in blood pressure, heart rate and body mass of physically active men in response to thermal stresss. Cent Eur J Sport Sci Med. (2022) 37:65–76.

[46] Aandstad A Holtberget K Hageberg R Holme I Anderssen SA. Validity and reliability of bioelectrical impedance analysis and skinfold thickness in predicting body fat in military personnel. Mil Med. (2014) 179:208–17. doi: 10.7205/MILMED-D-12-00545, PMID: 24491619

[47] Boraczyński T Boraczyński M Podstawski R Borysławski K Jankowski K. Body mass loss in dry sauna and heart rate response to heat stress. Rev Bras Med Esporte. (2018) 24:258–62. doi: 10.1590/1517-869220182404172175

[48] Podstawski R Borysławski K Clark CCT Choszcz D Finn KJ Gronek P. Correlations between repeated use of dry sauna for 4 x 10 minutes, physiological parameters, anthropometric features, and body composition in young sedentary and overweight men: health implications. Biomed Res Int. (2019) 2019:7535140. doi: 10.1155/2019/7535140, PMID: 30800676 PMC6360547

[49] Podstawski R Borysławski K Laukkanen JA Clark CCT Choszcz D. The effect of prolonged thermal stress on physiological parameters of young, sedentary men and the correlations with somatic features and body composition parameters. Homo. (2019) 70:119–28. doi: 10.1127/homo/2019/1016, PMID: 31475725

[50] Searight H. R. Montone K. (2020). Profile of mood states. Encycl Personal Individ Differ. 4057–4062. Available at: https://en.wikipedia.org/wiki/Sensitivityandspecificity (Accessed June 2, 2021).

[51] Lee J Park BJ Tsunetsugu Y Ohira T Kagawa T Miyazaki Y. Effect of forest bathing on physiological and psychological responses in young Japanese male subjects. Public Health. (2011) 125:93–100. doi: 10.1016/j.puhe.2010.09.005, PMID: 21288543

[52] Podstawski R Finn KJ Borysławski K Omelan AA Podstawska AM Skrzypczak AR. The influence of COVID-19 on university students’ well-being, physical activity, body composition, and strength endurance. Int J Environ Res Public Health. (2022) 19:15680. doi: 10.3390/ijerph192315680, PMID: 36497754 PMC9740601

[53] Gifford RM Todisco T Stacey M Fujisawa T Allerhand M Woods DR. Risk of heat illness in men and women: a systematic review and meta-analysis. Environ Res. (2019) 171:24–35. doi: 10.1016/j.envres.2018.10.020, PMID: 30641370

[54] Center for Disease Control and Prevention, (2023). Warning signs and symptoms of heat-related illness. Available at: https://www.cdc.gov/disasters/extremeheat/warning.html (Accessed July 23, 2022).

[55] Shin SJ Yoo H Park MC. New type of sauna-related burn: conductive contact burn. J Craniofac Surg. (2013) 24:e48–50. doi: 10.1097/SCS.0b013e31826d08fd, PMID: 23348336

[56] Huang G Shi X Davis-Brezette JA Osness WH. Resting heart rate changes after endurance training in older adults: a meta-analysis. Med Sci Sports Exerc. (2005) 37:1381–6. doi: 10.1249/01.mss.0000174899.35392.0c, PMID: 16118586

[57] Fox SM III Naughton JP Haskell WL. Physical activity and the prevention of coronary heart disease. Ann Clin Res. (1971) 3:404–32. PMID: 4945367

[58] Oja P Tuxworth B. Eurofit for adults. Assessment of health-related fitness: Tampere, Council of Europe Publishing (1995).

[59] Piątkowska M Biernat E. Participation and reasons for non-participation in sport and recreational activities before and after the outbreak of COVID-19: analysis of data from the 2016 and 2021 Poland National Sports Participation Survey. Phys Cult Sport Stud Res. (2023) 101:63–76. doi: 10.2478/pcssr-2023-0025

[60] Franklin BA Rusia A Haskin-Popp C Tawney A. Chronic stress, exercise and cardiovascular disease: placing the benefits and risks of physical activity into perspective. Int J Environ Res Public Health. (2021) 18:9922. doi: 10.3390/ijerph18189922, PMID: 34574843 PMC8471640

[61] Haseba S Sakakima H Kubozono T Nakao S Ikeda S. Combined effects of repeated sauna therapy and exercise training on cardiac function and physical activity in patients with chronic heart failure. Disabil Rehabil. (2016) 38:409–15. doi: 10.3109/09638288.2015.1044032, PMID: 25941983

[62] Kihara T Biro S Ikeda Y Fukudome T Shinsato T Masuda A. Effects of repeated sauna treatment on ventricular arrhythmias in patients with chronic heart failure. Circ J. (2004) 68:1146–51. doi: 10.1253/circj.68.1146, PMID: 15564698

[63] Miyamoto H Kai H Nakaura H Osada K Mizuta Y Matsumoto A. Safety and efficacy of repeated sauna bathing in patients with chronic systolic heart failure: a preliminary report. J Card Fail. (2005) 11:432–6. doi: 10.1016/j.cardfail.2005.03.004, PMID: 16105634

[64] Sawicka A Brzostek T Kowalski R. Effects of sauna bath on the cardiovascular system. Med Rehabil. (2007) 11:15–22.

[65] Leicht AS Halliday A Sinclair WH D’Auria S Buchheit M Kenny DP. Heart rate variability response to acute, and repeated pos-exercise sauna in trained cyclists. Appl Physiol Nutr Metab. (2018) 43:704–10. doi: 10.1139/apnm-2017-0581, PMID: 29444412

[66] Hasan J Karvonen MJ Pirronen P. Special review. II. Physiological effects of extreme heat. As studied in the Finnish “sauna” bath. Am J Phys Med. (1967) 46:1226–46. PMID: 5337808

[67] Ketelhut S Ketelhut RG. The blood pressure and heart rate during sauna bath correspond to cardiac responses during submaximal dynamic exercise. Complement Ther Med. (2019) 44:218–222. doi: 10.1016/j.ctim.2019.05.002 31126559

[68] Keast ML. The Finnish sauna bath and its use in patients with cardiovascular disease. J Cardpulm Rehabil. (2000) 20:225–30. doi: 10.1097/00008483-200007000-00002 10955262

[69] Podstawski R Boraczyński T Boraczyński M Choszcz D Mańkowski S Markowski P. Sauna-induced body mass loss in young sedentary women and men. Sci World J. (2014) 2014:307421:1–7. doi: 10.1155/2014/307421, PMID: 25614882 PMC4295591

[70] Pilch W Szyguła Z Torii M. Effect of the sauna-induced thermal stimuli of various intensity on the thermal and hormonal metabolism in women. Biol Sport. (2007) 24:357–73.

[71] Hussain J Cohen M. Clinical effects of regular dry sauna bathing: a systematic review. Evid Based Complement Alternat Med. (2018) 2018:1857413–30. doi: 10.1155/2018/1857413, PMID: 29849692 PMC5941775

[72] Barwood MJ Goodall S Bateman J. The effect of hot and cold drinks on thermoregulation, perception, and performance: the role of the gut in thermoreception. Eur J Appl Physiol. (2018) 118:2643–54. doi: 10.1007/s00421-018-3987-8, PMID: 30203296

[73] LaFata D Carlson-Phillips A Sims ST Russell EM. The effect of a cold beverage during an exercise session combining both strength and energy systems development training on core temperature and markers of performance. J Int Soc Sports Nutr. (2012) 9:44. doi: 10.1186/1550-2783-9-44, PMID: 22992430 PMC3472188

[74] Masuda A Koga Y Hattanmaru M Minagoe S Tei C. The effects of repeated thermal therapy for patients with chronic pain. Psychother Psychosom. (2005) 74:288–94. doi: 10.1159/000086319 16088266

[75] Hussain JN Greaves RF Cohen MM. A hot topic for health: results of the global sauna survey. Complement Ther Med. (2019) 44:223–34. doi: 10.1016/j.ctim.2019.03.012, PMID: 31126560

[76] Brunt VE Eymann TM Francisco MA Howard MJ Minson CT. Passive heat therapy improves cutaneous microvascular function in sedentary humans via improved nitricoxide-dependent dilation. J Appl Physiol. (2016) 121:716–23. doi: 10.1152/japplphysiol.00424.2016, PMID: 27418688 PMC6195670

[77] Carabaño MJ Logar B Bormann J Minet J Vanrobays ML Díaz C. Modeling heat stress under different environmental conditions. J Dairy Sci. (2016) 99:3798–814. doi: 10.3168/jds.2015-10212 26923054

[78] Nag PK Nag A Ashtekar SP. Thermal limits of men in moderate to heavy work in tropical farming. Ind Health. (2007) 45:107–17. doi: 10.2486/indhealth.45.107, PMID: 17284882

[79] Yang FL Lee CC Subeq YM Lee CJ Ke CY Lee RP. Heat adaptation from regular hot water immersion decreases proinflammatory responses, HSP70 expression, and physical heat stress. J Therm Biol. (2017) 69:95–103. doi: 10.1016/j.jtherbio.2017.06.012, PMID: 29037410

[80] Morrison SF. Central neural control of thermoregulation and brown adipose tissu. Auton Neurosci. (2016) 196:14–24. doi: 10.1016/j.autneu.2016.02.010 26924538 PMC4846468

[81] Périard JD Travers GJ Racinais S Sawka MN. Cardiovascular adaptations supporting human exercise-heat acclimation. Auton Neurosci. (2016) 196:52–62. doi: 10.1016/j.autneu.2016.02.002, PMID: 26905458

[82] Ely BR Lovering AT Horowitz M Minson CT. Heat acclimation and cross tolerance to hypoxia: bridging the gap between cellular and systemic responses. Temperature (Austin). (2014) 1:107–14. doi: 10.4161/temp.29800, PMID: 27583292 PMC4977168

[83] Mee JA Peters S Doust JH Maxwell NS. Sauna exposure immediately prior to short-term heat acclimation accelerates phenotypic adaptation in females. J Sci Med Sport. (2018) 21:190–195. doi: 10.1016/j.jsams.2017.06.024 28739443

[84] Collier RJ Gebremedhin KG. Thermal biology of domestic animals. Annu Rev Anim Biosci. (2015) 3:513–32. doi: 10.1146/annurev-animal-022114-110659 25387108

[85] Notley SR Park J Tagami K Ohnishi N Taylor NAS. Variations in body morphology explain sex differences in thermoeffector function during compensable heat stress. Exp Physiol. (2017) 102:545–62. doi: 10.1113/EP086112, PMID: 28231604

[86] Wong BJ Hollowed CG. Current concepts of active vasodilation in human skin. Temperature. (2017) 4:41–59. doi: 10.1080/23328940.2016.1200203, PMID: 28349094 PMC5356216

[87] Koene RJ Adkisson WO Benditt DG. Syncope and the risk of sudden cardiac death: evaluation, management, and prevention. J Arrhythm. (2017) 33:533–44. doi: 10.1016/j.joa.2017.07.005, PMID: 29255498 PMC5728985

